# A Smart Glass Telemedicine Application for Prehospital Communication: User-Centered Design Study

**DOI:** 10.2196/53157

**Published:** 2024-11-29

**Authors:** Zhan Zhang, Enze Bai, Yincao Xu, Aram Stepanian, Jared M Kutzin, Kathleen Adelgais, Mustafa Ozkaynak

**Affiliations:** 1 School of Computer Science and Information Systems Pace University New York, NY United States; 2 Icahn School of Medicine Mount Sinai New York, NY United States; 3 Mount Sinai Hospital New York, NY United States; 4 School of Medicine University of Colorado Aurora, CO United States; 5 Children's Hospital Colorado Aurora, CO United States; 6 College of Nursing University of Colorado Aurora, CO United States

**Keywords:** smart glass, telemedicine, participatory design, emergency medical service, health care, prehospital care, mobile health, mHealth, augmented reality

## Abstract

**Background:**

Smart glasses have emerged as a promising solution for enhancing communication and care coordination among distributed medical teams. While prior research has explored the feasibility of using smart glasses to improve prehospital communication between emergency medical service (EMS) providers and remote physicians, a research gap remains in understanding the specific requirements and needs of EMS providers for smart glass implementation.

**Objective:**

This study aims to iteratively design and evaluate a smart glass application tailored for prehospital communication by actively involving prospective users in the system design process.

**Methods:**

Grounded in participatory design, the study consisted of 2 phases of design requirement gathering, rapid prototyping, usability testing, and prototype refinement. In total, 43 distinct EMS providers with diverse backgrounds participated in this 2-year long iterative design process. All qualitative data (eg, transcribed interviews and discussions) were iteratively coded and analyzed by at least 2 researchers using thematic analysis. Quantitative data, such as System Usability Scale (SUS) scores and feature ratings, were analyzed using statistical methods.

**Results:**

Our research identified challenges in 2 essential prehospital communication activities: contacting online medical control (OLMC) physicians for medical guidance and notifying receiving hospital teams of incoming patients. The iterative design process led to the identification of 5 key features that could potentially address the identified challenges: video call functionality with OLMC physicians, call priority indication for expedited OLMC contact, direct communication with receiving hospitals, multimedia patient information sharing, and touchless interaction methods for operating the smart glasses. The SUS score for our system design improved from a mean of 74.3 (SD 11.3) in the first phase (classified as *good* usability) to 80.3 (SD 13.1) in the second phase (classified as *excellent* usability). This improvement, along with consistently high ratings for other aspects (eg, willingness to use and feature design), demonstrated continuous enhancement of the system’s design across the 2 phases. Additionally, significant differences in SUS scores were observed between EMS providers in urban areas (median 85, IQR 76-94) and rural areas (median 72.5, IQR 66-83; Mann-Whitney *U*=43; *P*=.17), as well as between paramedics (median 72.5, IQR 70-80) and emergency medical technicians (median 85, IQR: 74-98; Mann-Whitney *U*=44.5; *P*=.13), suggesting that EMS providers in urban settings and those with less training in treating patients in critical conditions perceived the smart glass application as more useful and user-friendly. Finally, the study also identified several concerns regarding the adoption of the smart glass application, including technical limitations, environmental constraints, and potential barriers to workflow integration.

**Conclusions:**

Using a participatory design approach, this study provided insights into designing user-friendly smart glasses that address the current challenges EMS providers face in dynamic prehospital settings.

## Introduction

### Background

Emergency medical services (EMSs) are a specialized medical domain dedicated to providing urgent medical care to patients who are critically ill or the place where an incident occurred. It involves dispatching certified emergency care clinicians, such as paramedics and emergency medical technicians (EMTs), to the scene of an emergency. The primary objective of EMS providers is to promptly stabilize and treat patients with complex and constantly changing conditions. Furthermore, EMS providers must dedicate time to collecting, sharing, and discussing crucial patient information with remote care teamsa process known as *prehospital communication*.

Effective and accurate prehospital communication is critical for ensuring that remote care teams (eg, emergency department [ED] physicians and nurses at the receiving facility) understand the patients’ conditions and are thus able to offer more accurate guidance, better prepare for the patient’s arrival, and mobilize necessary resources [1]. In the current practice, EMS providers rely on a radio or a phone to communicate with remote care teams. However, these conventional communication methods pose challenges not only for EMS providers trying to verbally convey complex patient situations but also for remote care teams attempting to comprehend the patients’ status. Therefore, previous research has called for more advanced technology solutions to address the inherent limitations of conventional communication methods [2,3]. Recognizing this critical need, researchers have explored the potential of telemedicine systems to facilitate prehospital communication [4-11]. Notable examples include ambulance-based telemedicine systems, where computers and cameras are installed inside ambulances to capture video footage of designated areas (eg, the patient body), which can be streamed to remote physicians for medical guidance or decision support. Although these systems can significantly enhance communication and information sharing during EMS care and transport, their adoption rate remains low due to various limitations (eg, lack of portability, limited usability, and reliance on manual input and control) [5,11,12]. For example, Cho et al [5] reported that the size and weight of the telemedicine unit made it cumbersome or even impossible to use outside the ambulance, where a great portion of patient care takes place. In addition, manually handling these systems increases the risk of cross contamination and patient infections [13]. These limitations of ambulance-based telemedicine systems hinder their effective use in the dynamic, mobile, and hands-on EMS environment.

Given the limitations of ambulance-based telemedicine systems, there is a need for more portable telemedicine technologies that can accommodate the hands-busy and mobile nature of EMS work. Smart glasses have emerged as a promising solution due to their potential advantages, such as high portability and hands-free operation [14,15]. For example, several studies have tested the affordances and feasibility of using smart glasses to enable real-time sharing of visual medical information from the field with remote emergency physicians [14,16-18]. In this existing body of research, most studies have used off-the-shelf smart glass devices and teleconferencing software for testing purposes, such as evaluating the technical feasibility or usability of these devices in EMS work [19]. Yet, these studies did not engage with EMS providers to identify their specific needs or to gather their perspectives on how the smart glasses should be designed to align with the unique characteristics of EMS work [20,21]. Therefore, a critical research question remains unanswered: How can smart glasses be designed as a telemedicine platform to enhance prehospital communication while considering the hands-busy and time-critical nature of EMS work?

To address this research question, we adopted a user-centered, participatory design (PD) approach to ensure the involvement of prospective users in the smart glass design process. This work is part of a larger research effort aimed at designing and developing an integrated telemedicine system to enhance prehospital communication, which includes smart glasses worn by EMS providers to connect with a separate desktop application used by remote emergency physicians or experts. Our design goals are not to replace current communication systems (eg, radio) but rather to supplement them to address some of the long-lasting challenges in the prehospital communication process. In this paper, we specifically focused on presenting a detailed account of our 2-year long user-centered design process for the smart glass application.

This work made the following contributions to the fields of medical and health care informatics: (1) an empirical understanding of the challenges and barriers in prehospital communication; (2) design insights for implementing smart glass–based telemedicine systems tailored for fast-paced, hands-busy medical teams such as EMS; and (3) user perceptions and potential barriers about the adoption and use of smart glasses in time-critical medical scenarios.

### Related Work

#### PD in Medical Work

The PD methodology facilitates the rapid development and evaluation of design concepts by directly involving intended users in the development of information systems [22,23]. This approach has proven effective in designing and developing health information technologies (HITs) [24], ensuring their efficiency, user-friendliness, and seamless integration into clinical workflow [25-28].

Several prior studies have used PD approaches to design and develop HITs for emergency care providers. For example, Kusunoki et al [29,30] engaged ED providers in PD workshops to co-design information displays that support awareness and enhance ED teamwork. Østervang et al [31] conducted PD workshops with patients, family members, health care professionals, and IT specialists to co-design a patient health information system. Another highly relevant work by Kristensen et al [22] used PD to understand the nuanced practices in prehospital care workflow and generate technology ideas and concepts for future EMS practice. Building on this body of work, we used the PD approach in our study to explore how smart glasses should be designed specifically for the prehospital care setting, an area previously unexplored using the PD method.

#### Smart Glasses in Health Care

The hands-free capabilities of smart glasses allow health care providers to use both hands for patient care while accessing, viewing, and sharing patient information at the point of care. Studies have shown how smart glasses can enhance collaboration among health care providers in different locations, such as when a local surgeon uses smart glasses to receive real-time guidance from a remote expert [32,33], or a nursing student can get support from an expert during cardiopulmonary resuscitation [34]. Particularly relevant to our work are studies investigating the feasibility and potential of using smart glasses in prehospital care settings [19]. In these studies, EMS providers used smart glasses to share live visual medical data from the field with remote emergency physicians, facilitating immediate and informed medical decisions [14,16-18]. These studies highlight the valuable role that smart glasses can play in enhancing communication and coordination between prehospital teams and remote emergency physicians or experts.

However, most of this research has relied on commercially available devices without incorporating feedback from end users in the design process. This gap could result in a mismatch between the technology design and the actual needs and workflows of medical professionals, potentially hindering the adoption of new HITs in complex medical settings [35]. Therefore, it is essential to understand how to effectively integrate smart glasses into the prehospital communication workflow while considering their dynamic and hands-on care practices. To address this gap, this study adopted a PD methodology, engaging EMS providers in an iterative design and evaluation process to create a smart glass application that meets their specific requirements and workflow.

## Methods

### Study Design

**Figure 1:**
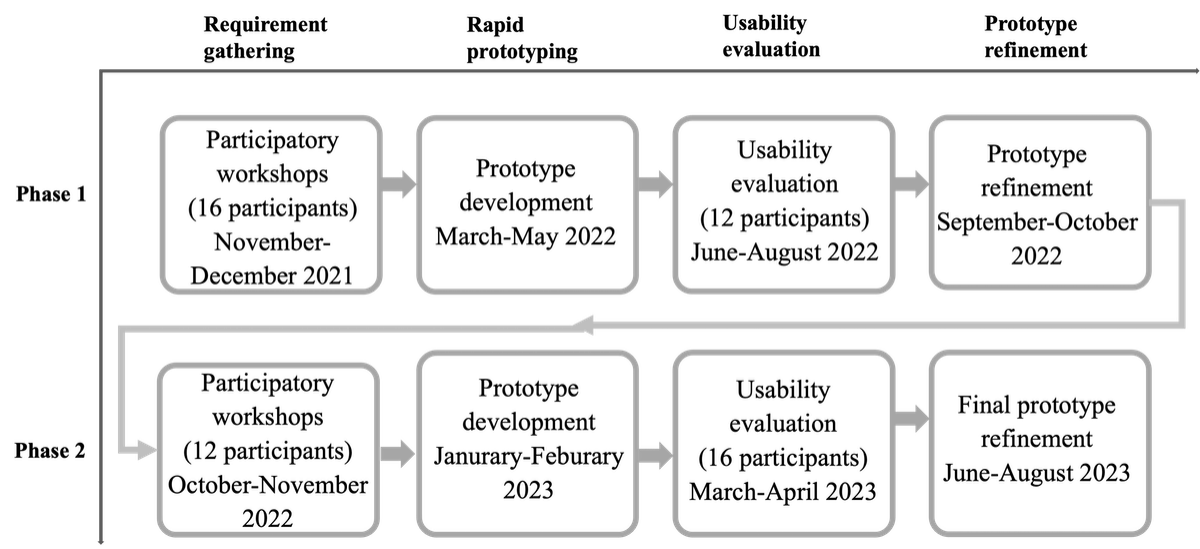
An illustration depicting the 2-year user-centered design study process.

In this study, we used an iterative user-centered design approach, combining PD [22,23] with usability evaluation [36,37]. This study comprised 2 phases, each including design requirement gathering, rapid prototyping, usability evaluation, and prototype refinement (Figure 1). The entire study lasted from November 2021 to April 2023.

We recruited study participants from 4 EMS agencies: 1 fire-based agency located in the rural mountain region of the United States and 3 hospital-based EMS agencies in an urban area on the East Coast of the United States. The diverse characteristics of these participating EMS agencies (eg, fire vs hospital-based agencies and urban vs rural settings) enhance the generalizability of our research. Directors at each EMS agency disseminated a recruitment email to their teams, instructing interested members to contact the researcher directly. The research objective and eligibility criteria for participation were explained in the recruitment email. The only eligibility criterion for participation was being a licensed EMS provider in those agencies, regardless of gender, ethnicity, years of experience, or other factors. Upon receiving responses from EMS providers, the researchers coordinated with each participant to schedule a convenient time for their involvement in the study. In addition, researchers answered any questions that providers had about the project and offered further details when requested.

In total, 43 unique EMS providers participated in our study, with 8 (19%) of them taking part in >1 session. The recruited participants held different roles (eg, paramedic vs EMT) and had a wide range of experience (from <1 year to >40 years). More specifically, 20 (47%) participants were paramedics, while the rest (23/43, 53%) were EMTs; 13 (30%) were recruited from the rural area-based agency, with the rest (30/43, 70%) from the urban-based agencies. Participant demographics and the sessions they attended are detailed in Multimedia Appendix 1.

### Data Collection

#### PD Workshop

We conducted 4 PD workshops in phases 1 and 3 PD workshops in phase 2, with each workshop including 4 participants. The number of participants for each phase and the timing of the workshops in each phase can be found in Figure 1.

During phase 1, our primary focus was to understand the challenges of the prehospital communication process, identify critical system features catering to the needs of EMS providers, and determine the most preferred interaction methods for using the smart glass device. In phase 2, the research team shifted its focus to refining system designs and features and identifying various factors and barriers that could affect the practical application of smart glasses in real-world scenarios.

Each workshop lasted up to 2 hours and included the following activities: (1) group discussions, where participants engaged in group discussions to share their requirements and suggestions for creating a smart glass–based telemedicine system; (2) individual sketching, where each participant had the opportunity to either create different design concepts through paper sketching and explain their design rationale or critique an existing prototype created in a previous phase, (3) group-based design, where participants collectively refined and developed design concepts that everybody agreed upon [22,29,30,38,39], and (4) wrap-up discussion, where participants shared their insights regarding sociotechnical challenges in the effective adoption and use of smart glasses in their workflow. We have included the study protocol for the phase 1 workshop in Multimedia Appendix 2 to illustrate how we structured the workshops.

#### Prototyping and Usability Evaluation

**Figure 2:**
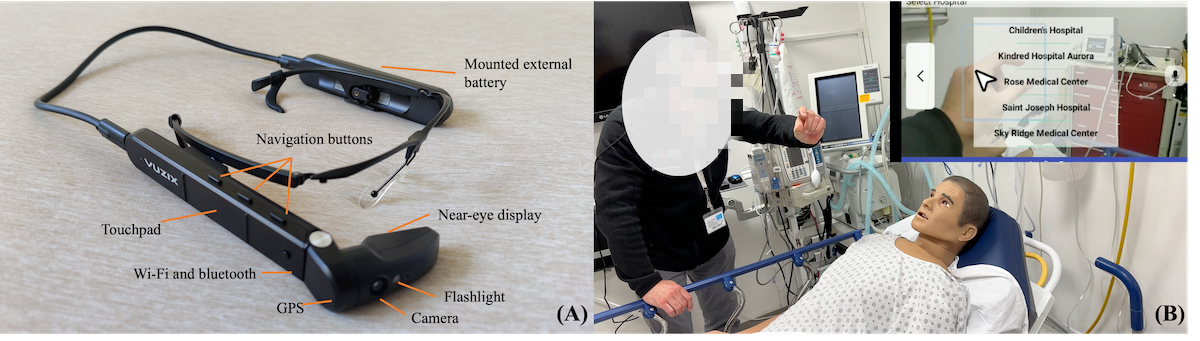
(A) The smart glass device (Vuzix M400) used in the project. (B) A study participant interacting with the application using hand gestures.

In each phase, the research team started the prototyping process by iteratively creating low-fidelity prototypes using paper and medium-fidelity prototypes using Figma (Figma Inc), based on the findings and user insights collected from preceding design or evaluation studies. Once the design (eg, system features) was finalized, we implemented functional prototypes using the Vuzix M400 platform (Vuzix Corporation), as depicted in Figure 2A. This device features a see-through, near-eye display that presents information without obstructing the wearer’s vision. It is also equipped with a camera adjacent to the near-eye display, enabling the capturing of still images and video streaming from a first-person point of view. The embedded GPS allows for real-time location tracking and sharing. By default, the device is operated through tangible buttons or a touchpad. The software development kit provided by Vuzix facilitates application development and enables the implementation of additional interaction methods (eg, voice commands). We used the Zoom application programming interface (Zoom Video Communications Inc) to develop video and audio call features and integrate them into our application. The prototype development in phase 1 took approximately 6 months due to several technical challenges. For example, the embedded Android system in our device as well as the gesture sensing software for implementing hands-free operation were using an old Native Developer Kit (NDK), whereas our teleconference software provider (Zoom) used a new NDK version. The use of different NDK versions of these software and hardware caused integration issues during compiling in a lower-level virtual machine. We worked closely with the technical support teams of all the companies, involving numerous communications and meetings, to resolve the issues of integrating different software packages.

After we successfully developed a functional system prototype, we conducted individual usability evaluations with EMS providers in a controlled environment, such as an office or a simulation laboratory (Figure 2B). The number of participants for each phase is depicted in Figure 1. We have included the study protocol for the usability evaluation in Multimedia Appendix 3 to illustrate how we structured the usability evaluation.

The purpose of this activity was to both gather design requirements and evaluate the designs. In particular, we were interested in finding out whether the application’s designs and features could effectively address the needs of EMS providers and current challenges in the prehospital communication process. At the beginning of each testing session, we provided training to the participants about system features and how to use the application. Once participants were confident enough to use the application independently, they were guided to complete various tasks corresponding to the major features of our application. This step involved participants using different interaction methods (such as tangible buttons and touchless interaction methods like voice commands) in a randomized order to perform an identical set of tasks. This approach helped us determine which interaction method was more user-friendly and practical for EMS providers when using the smart glass application to contact remote physicians.

We used both objective and subjective measures to evaluate the system’s usability. Objective measures included task completion time, task success rate, and errors encountered [40]. Subjective measures were administered through a survey, which consisted of a modified version of the System Usability Scale (SUS) [41] and a section of Likert-scale questions for participants to rate the usefulness of different system features and interaction methods on a scale of 1 to 5 (1 representing not useful at all and 5 representing very useful) [42-44], as well as a poststudy interview to elicit participants’ perceived benefits and challenges associated with using the application in practice. Feedback and responses from participants informed the prioritization of design modifications and prototype refinements in subsequent steps.

### Data Analysis

To facilitate data analysis, all research activities were both audio-recorded and videotaped. Photographs of the created artifacts (eg, design sketches or drawings) were also taken for data analysis purposes. The design sketches collected in design workshops and the annotations on paper prototypes gathered throughout the usability evaluations were used to understand how EMS providers envisioned the functionality and appearance of the application. Specifically, we combined these sketches and annotations with discussions on system features to understand not only the features participants wanted or envisioned but also how they would like to design the user flow and structure the screen layout for easy operation.

All discussions occurred in design workshops, and usability evaluations were transcribed, iteratively coded, and analyzed by at least 2 researchers using descriptive, thematic analysis [45-47]. The transcripts were managed and analyzed using NVivo (version 12; Lumivero). Our analysis focused on user requirements, feedback on system features, perceived benefits and concerns related to using smart glasses in the field, and the sociotechnical considerations of integrating smart glasses into EMS work. Two researchers began the qualitative data analysis by independently analyzing a small subset of transcripts and generating codebooks to standardize the data analysis process. We then used the Cohen κ coefficient to test interrater reliability by having the same 2 researchers independently code another small set of transcripts using the developed codebook and compare their codes. After achieving a substantial intercoder agreement, the 2 researchers separately coded the remaining transcripts. Any new codes that emerged through this process were added to the codebook. Disagreements in the analysis were discussed and resolved during weekly group meetings among all researchers. Once the coding process was completed, the generated codes were organized into high-level categories to identify overarching themes. The researchers also conducted member-checking to ensure the validity of data analysis. For instance, major findings from each study were presented to a subset of study participants and EMS agency directors to confirm that the researchers’ interpretations of the data accurately reflected providers’ opinions and real-world practices.

Descriptive statistical approaches were used to analyze quantitative data, such as SUS questionnaire responses and feature ratings by EMS providers. For instance, we followed the instructions as outlined by Brooke et al [41], to calculate the SUS score for each major design version and calculated the average rating given by participants for each feature. We also conducted the Mann-Whitney *U* test with Bonferroni corrected *P* values to assess differences in user ratings among participants with varying characteristics. More specifically, we compared SUS scores and user ratings of system features between EMTs and paramedics, EMS providers in rural areas versus urban areas, and those with <10 years of experience against those with >10 years of experience.

To evaluate the usability difference between tangible buttons and touchless interaction methods (eg, voice commands), we used both objective measurements (eg, task completion time, errors encountered, and the time taken to recover from errors) and subjective measurements (eg, user experiences collected through a questionnaire and posttest interviews). We applied the Friedman test to determine if significant differences in measurements (eg, task completion time and errors) existed across all interaction methods. If a significant difference was identified, we then conducted the Wilcoxon signed rank test with Bonferroni corrected *P* values for post hoc pairwise comparisons. More details about the assessment of usability differences between tangible buttons and touchless interaction methods can be found in the study by Zhang et al [48].

### Ethical Considerations

This research was approved by the Pace University institutional review board (1708685). All participants provided informed consent by reviewing and signing a consent form, which detailed their participation rights, the risks involved, and the fact that the study would be recorded. The research team also provided a verbal explanation of these rights, ensuring participants understood that their input would solely be used for research purposes and that their identities would be protected. To ensure anonymity and protect the privacy and confidentiality of the participants, all data collected were deidentified, with participant identities removed from transcripts or other data (eg, survey responses) and replaced by unique participant IDs. Finally, participants were compensated for their time at a rate of US $60 per hour. All participants took part in the research during their off-duty hours, ensuring that their normal patient care activities were not disrupted.The individual in Figure 2 provided written informed consent to allow their image to be published.

## Results

### Work Practices and Challenges in Prehospital Communication

#### Overview

In the first phase of PD workshops, we examined the current work practices and technology use of EMS providers as well as the challenges they frequently encounter during the prehospital communication process. Understanding these aspects is crucial for designing a smart glass application that effectively tackles the challenges in prehospital communication and aligns with the needs of EMS providers. We subsequently describe the 2 prominent activities in the prehospital communication process and the challenges associated with them.

#### Call Online Medical Control Physicians for Medical Guidance

**Figure 3:**
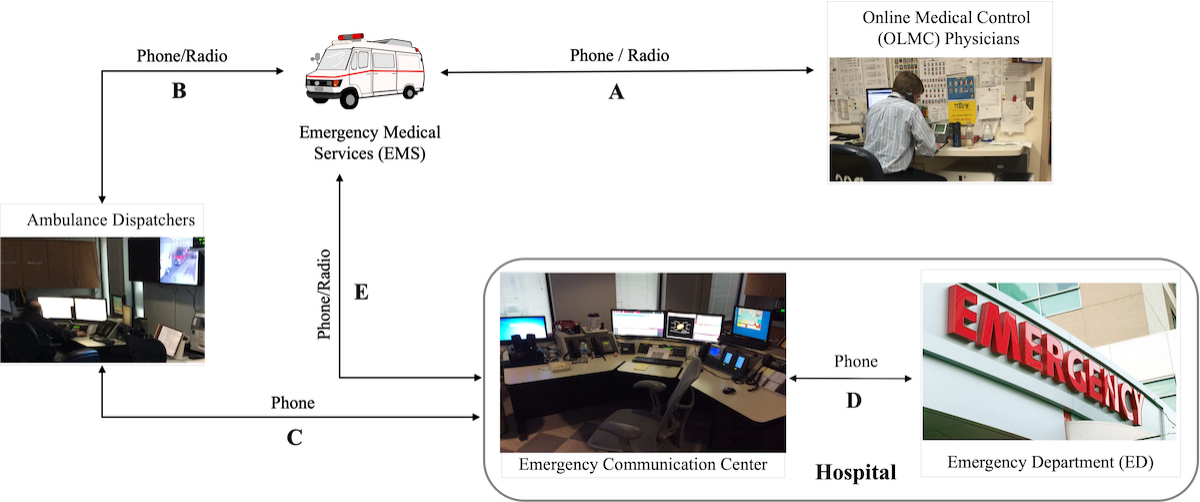
A typical prehospital communication process. (A) Emergency medical service (EMS) contacts online medical control physicians to receive medical guidance. (B) EMS calls ambulance dispatchers to initiate prehospital communication. (C) Ambulance dispatchers route the EMS call to the designated hospital. (D) The hospital’s dedicated emergency communication center receives and relays prehospital information to the emergency department. (E) EMS providers may also call the hospital directly to expedite the notification process.

A crucial prehospital communication activity for EMS providers is contacting an online medical control (OLMC) physician to seek medical guidance and oversight (Figure 3A), a process guided by established protocols. Reasons for contacting OLMC physicians include seeking approval for medication administration and obtaining decision support regarding patient destination. The OLMC system can be either centralized, with physicians operating from a centralized office, or decentralized, allowing physicians to operate from any location using a dedicated phone or application. In our study, the urban area on the East Coast used a centralized OLMC system design, while the rural area in the mountain region adopted a decentralized design.

The most significant challenge encountered when contacting the OLMC physician was the prolonged waiting time due to the physicians’ limited capacity to handle a large volume of EMS calls. In the urban area where 3 of our study sites were located, only 2 physicians were on call to handle incoming calls from EMS teams serving millions of people. This situation imposed an immense workload on OLMC physicians, causing significant delays in responding to EMS calls. Nearly all our study participants expressed frustration with the current OLMC system:

In our city, all five boroughs call one physician. So if that one physician is handling five, six, or seven calls, you could be on hold. Depending on the priority of your patient and what you need, I’ve been on hold [for] 45 minutes to an hour at times.

Participant 8

Another participant shared a similar experience, emphasizing that prolonged waiting times to connect with an OLMC physician are not uncommon, even in critical scenarios:

Even in a cardiac arrest, maybe we want orders for extra medications, or just to report the time of death, or maybe we want to transfer the patient out of there, we continue with our CPR and administer medicine while waiting on the phone to determine the next steps.

Participant 5

The challenge of communicating with OLMC physicians is exacerbated by the use of radios, as it complicates the sharing of contextual information (eg, patient status and symptoms), leading to miscommunications between EMS providers and OLMC physicians:

Miscommunication is a constant issue. It’s just an outdated form of communication, it’s what we feel about the radio we’re using.

Participant 9

#### Notify Hospital for Incoming Patients

In addition to contacting OLMC physicians for medical guidance, EMS providers must also inform the receiving hospital about the patient being transported to their facility. This notification is typically succinct but includes crucial details such as the patient’s age, symptoms, initial impression, treatments administered, and estimated time of arrival (ETA). This vital information allows the receiving hospital team, including physicians and nurses in the ED, to anticipate the patient’s needs and allocate necessary resources promptly.

Similar to contacting OLMC physicians, the primary issue with hospital notification is the ineffective communication arising from radio use, as highlighted in prior studies [2,3]. Our participants expressed similar concerns about relying exclusively on radios for hospital notification:

So when EMS providers [are] in the field, they find that it’s very time consuming to tell the hospital team about the [patient’s] stats. Miscommunicated patient stats are a problem that’s faced [by care teams]. There’s a lot of contextual information that gets lost because the radio is an old piece of technology.

Participant 9

Furthermore, as depicted in Figure 3, EMS providers must navigate several communication layers when attempting to notify the receiving ED team. Typically, EMS providers begin the communication process by contacting their dispatcher (Figure 3B), who then transfers the call to the designated hospital (Figure 3C). Some hospitals have an emergency communication center specifically for managing calls from EMS providers and conveying patient information to the ED team (Figure 3D). This multilayer communication approach often results in miscommunication or delays in hospital notification [1]. For instance, in urban cities where transport time is generally shorter compared to rural areas, the ambulance might arrive at the hospital before the ED team receives a notification, as 1 participant explained:

I think the biggest problem we have is that, at least here in our city, we have very short timeframes. We’re talking about five minutes to get to the closest trauma center or thrombectomy center. And sometimes when we get to the hospital, and they [ED providers] don’t even know why we are there because the notification hasn’t been sent through yet. That’s, you know, because you have to go through different people to get the message along. Also, because we are using radios and it takes several times for the radio transmission to go through.

Participant 10

To circumvent these problems, several participants revealed a work-around they have been using, that is, if they transport the patient to their affiliated hospital, they call the ED directly using their personal phones to expedite the notification process (Figure 3E).

### Iterative Design of a Smart Glass Application for Addressing Challenges in the Prehospital Communication Process

#### Overview

**Figure 4:**
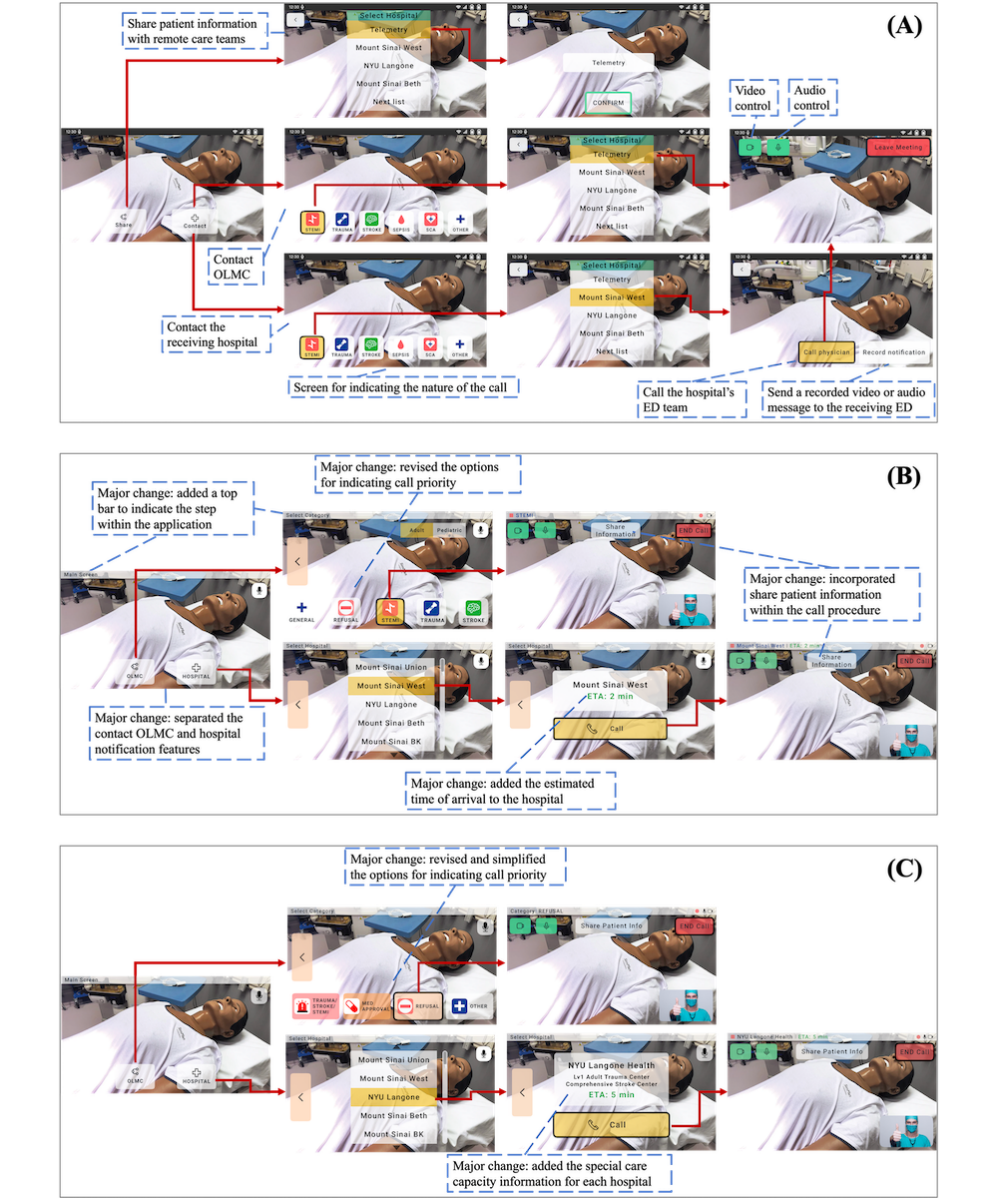
Iterative design of the smart glass application. (A) The first major application version created and tested in phase 1. (B) The second major application version created and tested in phase 2. (C) The final design of the smart glass application. ED: emergency department; ETA: expected time of arrival; OLMC: online medical control.

Our iterative, user-centered design and evaluation process resulted in the creation of >20 design versions, with 2 major iterations evaluated in the first and second phases of usability testing. In this section, we focus on detailing the most necessary system features, as well as those explored but deemed unnecessary or unhelpful by EMS providers. Figure 4 presents the 2 major versions evaluated through usability testing and the final design of our application, with added notes in the figure to highlight significant design changes in each iteration.

#### Most Needed System Features by EMS Providers

##### Overview

Through various design and evaluation studies, EMS providers highlighted five crucial system features that could improve the current prehospital communication process: (1) establishing video calls with OLMC physicians, (2) indicating call priority for expeditious OLMC contact, (3) enabling direct EMS-ED communication, (4) sharing multimedia patient information with remote care teams, and (5) enabling touchless device operation.

##### Establishing Video Calls With OLMC Physicians

Across all study sessions, there was a consensus among EMS providers that smart glasses could be invaluable for consulting with OLMC physicians through video calls, enabling physicians to see exactly what EMS providers see. This feature could significantly enhance physicians’ understanding of the patient’s condition and needs, leading to more informed decision-making. Participants consistently expressed high satisfaction with this feature, rating it an average of 4.45 (out of 5) in phase 1 usability studies and an even higher 4.75 in phase 2 studies. One participant explained as follows:

So being able to get on the scene and use smart glasses and being able to say, ‘well, this person really doesn’t need an ambulance, or they could go to a clinic instead, or there’s something very serious,’ the doctor can, you know, can better help out.

Participant 1

On the basis of user feedback, we incorporated an intuitive, easy-to-operate video call feature for contacting OLMC, allowing EMS providers to turn the camera and audio on or off and leave the call.

Our initial design combined the options to contact OLMC and nearby hospitals into 1 list, with the OLMC option always focused and placed at the top (Figure 4A). This design intended to consolidate both OLMC contact and hospital notification on 1 screen for convenience and easy access. However, subsequent studies (eg, usability testing) prompted a significant design modification, that is, separating “Call OLMC” and “Notify Hospital” from the same list into different screens (Figure 4B). This change was driven by 2 primary considerations. First, EMS providers typically contact hospitals more frequently than OLMC physicians; positioning OLMC as the first option in the list could lead to extraneous device operations as EMS providers would always have to click multiple times to navigate down the list to find the hospital. Second, as described subsequently, our application includes a screen allowing EMS providers to indicate call priority. Participants noted that specifying call urgency was not necessary before connecting with the receiving hospital. Unlike contacting OLMC physicians, who need to prioritize which call to take first, the receiving hospital typically does not need to know the call urgency before picking up the EMS call. Participants in phase 2 usability testing confirmed that it is not necessary to indicate call priority when contacting the hospital, leading us to keep this revised design in the final version (Figure 4C).

##### Indicating Call Priority to Expedite OLMC Connection

Given the persistent issue of lengthy waiting times to connect with OLMC physicians, we designed a feature that allows EMS providers to indicate the urgency of the call. One participant highlighted the usefulness of this feature by stating the following:

It [smart glasses] can be used to solve a bigger problem in terms of the congestion of actually triaging patients with online medical control, which I would really like to highlight here. You could really solve those problems using this technology.

Participant 3

This feature underwent significant design changes across the 2 phases. As shown in Figure 4A, the initial design included categories for critical patient conditions such as “STEMI” (ST elevation myocardial infarction), “trauma,” “stroke,” “sepsis,” and “SCA” (Sudden Cardiac Arrest). However, participants felt that limiting the call nature option to only critical scenarios could render it less useful, as all such scenarios have comparable urgency:

Prioritize. I think having just a critical or uncritical option to pick between is probably going to be more useful than having many critical options.

Participant 19

In addition, a few participants also saw the value in distinguishing between adult and pediatric patients, leading to a major design revision introducing a toggle to specify the patient type. We then refined the options within each type to reflect call priority. For instance, in the adult patient category, we included categories such as “general” and “refusal” for nonurgent consultations and categories such as “trauma,” “stroke,” and “STEMI” for critical conditions (Figure 4B). The “general” category represents nonurgent reasons for contacting OLMC, such as requests for permission for additional medication doses, and “refusal” pertains to situations where physician approval is needed when patients refuse medical attention. These revisions aimed to enhance distinctions between critical and noncritical scenarios and between adult and pediatric cases.

During phase 2 usability testing, feedback was received that the process of selecting between adult and pediatric patients and then choosing the nature of the call was overly complex. As a result, we refined the design to simplify user interaction by removing the required step to select between adult and pediatric patients. Participants also suggested revisions to the options for indicating call priority:

What I would suggest under the medical control would be a general alert like for medication approval. You know, you’re gonna have a lot of stuff that’s gonna fit under there. And then a separate refusal category. And lastly, a category combining STEMI, traumas, and stroke, like those big ones. So yeah, I think these pretty much have most cases covered.

Participant 40

We then followed their suggestions to include 4 options adaptable to different patient types: “medication approval” and “refusal” for common reasons for contacting OLMC; “trauma/stroke/STEMI” for conditions requiring immediate attention and prioritization by OLMC; and an “other” category for other types of or nonurgent issues (Figure 4C). To visually delineate the urgency of calls, different colors were used, ranging from red for critical conditions to white for the “other” category.

##### Facilitating Direct EMS-ED Communication

Through our research, it became evident that smart glasses can act as a valuable tool to facilitate direct communication between EMS and ED providers via video calls. This feature received consistently high usefulness ratings from participants (4.57 out of 5 in phase 1 of usability testing and 4.56 out of 5 in phase 2 of usability testing). One participant underscored that compared to relying solely on audio, having a direct video call with the ED physician or nurse could make it easier to describe the patient’s status, thus mitigating the risks of miscommunication and helping the receiving ED care team better understand the nature and needs of the incoming patients:

I really, really enjoyed how you can use this technology to report to the hospital. And you can actually do that much quicker [with smart glasses] than you would in real life. When we got on the scene, we can actually use smart glasses to easily describe what the actual scene looks like, if it’s going to be an actual trauma, how many patients they are going to have, etc. That could help the [emergency department of the receiving] hospital get prepared better.

Participant 3

As shown in Figure 4, EMS providers can conveniently select a hospital from a list organized by proximity to the current location of the EMS team. Using embedded GPS, the smart glasses can automatically calculate and display the ETA to the chosen hospital, a feature universally found useful by participants (Figures 4B and 4C):

I think the ETA to the hospital is very helpful. So I don’t have to look out the window of the ambulance and figure out my ETA to the hospital. It automatically tells that for me. So that way I can stay focused on my patient, instead of trying to figure out where my landmarks are.

Participant 12

Furthermore, during phase 2, participants suggested the inclusion of additional information for each hospital, such as the availability of trauma centers or pediatric care facilities, to aid EMS providers in choosing the most appropriate destination for patient needs:

Maybe you can put little icons next to each hospital name. So, who is a primary stroke center? Who is a primary trauma center? Who is a primary heart center? So, then they [EMS providers] can better determine which hospital to contact.

Participant 22

This addition is vital, as the nearest hospital might not always be equipped with specialized facilities (eg, a trauma center) to treat certain critical conditions. Following this suggestion, information regarding the specialized services available at each hospital was added to the final design (Figure 4C).

##### Sharing Multimedia Patient Information With Remote Care Teams

In phase 1 PD workshops, many of our participants emphasized the importance of sharing pivotal, textual, and visual patient information (eg, vital signs and photos) with remote care teams to facilitate their conversations. As 1 participant explained:

I don’t know if I can transmit the information directly to the hospital or online medical control through this type of technology. If so, that would be the absolute best thing that you could ever imagine.

Participant 4

To address this crucial user need, we initially designed a unique feature allowing information sharing independently of initiating contact with hospitals or OLMC (Figure 4A). This feature was deemed very useful and received an average rating of 4.6 (out of 5) in phase 1 usability testing.

Despite the positive view of this feature during phase 1, we discovered that sharing information with the hospital or OLMC before establishing the call could create a problematic workflow. The primary reason is that the remote care team may simply ignore the shared information if it comes before the call is established, as 1 participant explained:

If hospitals don’t have a dedicated person managing the shared information, then the usefulness of transmitting the information is limited. How would you transmit separately if you are not talking to the hospital?

Participant 1

On the basis of the received feedback, we made major changes to the design in phase 2. We integrated the feature of sharing patient information directly into the call feature, allowing EMS providers to share patient information only after the call is established (Figure 4B). This revised design received an average rating of 4.75 out of 5 during phase 2 usability testing, surpassing the rating received in phase 1. Given the overall positive feedback on this new design, we kept this design with minor wording modifications in our final version (Figure 4C).

##### Enabling Touchless Device Operation

Given the nature of EMS work, which often requires the use of hands and entails a risk of cross contamination, participants expressed a strong preference for touchless interaction methods to operate the smart glass device over using the default tangible buttons, as 1 participant explained:

So all hands being gloved up while we are taking care of a patient who’s bleeding. You can’t take your hand off when you’re doing compressions to activate the glasses. If you have blood on your gloves, you’re going to add it to the glasses. It will help so much if I don’t need to touch the glasses.

Participant 4

In phase 1 PD workshops, we explored the preferred interaction methods of EMS providers. Among a set of touchless interaction methods [49], voice commands and hand gestures emerged as the 2 most preferred choices. Of the 16 participants (1 participant did not submit the ranking), 10 (62%) indicated voice commands as their top choice, while 5 (31%) participants chose hand gestures as their most preferred method, with 10 (62%) considering it their second preference.

On the basis of these preferences, we integrated both voice commands and hand gestures into our system prototype. More specifically, we used the Vuzix Software Development Kit to program a set of simple voice commands corresponding to text labels on the virtual buttons. To implement the hand gesture–based interactions, we used third-party gesture-sensing software (CrunchFish). This interaction method allows users to perform a pinch gesture by tapping their thumb and index finger together to choose an option on the interface (Figure 2B).

In the usability testing studies conducted during both phases, we assessed the user experience and usability of voice commands and hand gestures compared to the default interaction method—tangible buttons (detailed results are reported in the study by Zhang et al [48]). A consistent finding across both phases of usability testing was that, compared to tangible buttons, voice commands and hand gestures had suboptimal task performance in all measured aspects (eg, task completion time and errors, etc). Despite this, our participants emphasized the importance and benefits of offering touchless interaction methods to minimize the need for physical operation of the smart glasses:

Because we’re always using our hands, I think voice commands in parallel with what we’re doing in the field are probably the best. And that’s been our biggest request for certain applications we use in the field because we really wish there was a voice command functionality where we can continue to interact with patient but using voice command for the computing device. So yes, that I think voice demand is probably the best.

Participant 43

I liked the hand gestures, because it’s very, it’s very intuitive and the connection between me and the interface felt the strongest.

Participant 19

#### Unnecessary System Features

##### Overview

Throughout the design and evaluation process, we explored various features and design concepts, some of which were deemed unnecessary. In this section, we report 2 features that were extensively discussed and tested but were considered unnecessary or not useful by our participants.

##### Recording Short Audio and Video Messages for Hospital Notification

In phase 1 PD workshops, a few EMS providers expressed interest in a feature allowing them to record and send a short audio or video message to notify the receiving hospital about incoming patients. This feature was believed to be especially useful when a direct connection with the receiving hospital could not be established. One participant highlighted its potential time-saving benefits:

I’m more concerned about notifications to the ED with an unstable or critical patient. And I’m not really consulting, because I’m not asking for any real orders. I’m giving them very short, very brief information. So, what could be helpful is maybe just record a message and then maybe just click “Send,” and then you could get a confirmation that the notification is delivered to your destination.

Participant 1

This user requirement informed our initial design to include a feature to deliver a prerecorded audio or video notification to the hospital ED team (Figure 4A).

However, this feature received mixed feedback during the usability testing studies in phase 1 and the following PD workshops in phase 2. While recognizing the potential time-saving benefits, EMS providers also raised concerns about new issues, such as providers opting not to call the hospital when they have the option to simply send a prerecorded message. There were also concerns about the receiving ED team overlooking the shared audio or video messages, potentially causing workflow disruptions and delays in preparation for patient arrival. For example, one participant explained his concern:

I don’t see this being very useful; I see it as more frustrating than anything else. Because if I’m doing the recording for notification, this person is probably actively dying in front of me. So if I want to give a notification [to the hospital], I just want to call them and I want to tell them this is what’s happening.

Participant 19

Given these concerns and the potential impact on current workflows, we decided to remove this feature from the prototype (Figures 4B and 4C).

##### Augmented Reality–Enabled Annotations

As the smart glass is powered by augmented reality technology, we explored the usefulness of enabling users to annotate real or virtual objects within their field of view. This would allow, for example, drawing a circle over a specific area on a patient’s body to draw the attention of remote physicians during teleconsultation. It could also enable the remote consultant to annotate images captured from the live stream and project them back into the visual field of the smart glass wearer for real-time guidance. This innovative feature has been implemented and tested in prior work [50,51].

However, most (12/16, 75%) of our participants in phase 1 workshops deemed the annotation feature unnecessary for 2 main reasons. First, it is very rare for EMS providers to seek guidance from remote physicians on performing a procedure; therefore, the need for annotations is very limited, as 1 participant explained:

That’s [using annotation] not likely going to happen. Because in order for us to be out in the field, we have to already have that training and those skills to be able to do that, whether it’s BLS [basic life support] or ALS [advanced life support] level. So doctors are not going to tell you, “Oh, start an IV on the left or on the right,” you know, paramedics already know. You know, if somebody is bleeding from a certain side, you don’t need a doctor to tell you “Hey, patch that,” you know that. I mean, I understand where you’re going with that, like giving more concrete instruction to a junior or not very experienced provider. But you can probably just verbalize anyway.

Participant 1

Second, using the annotation feature could become a distraction, as it requires extra operations with the device, such as selecting an annotation tool from a list of options and then closing the annotation toolbox:

As far as the annotation tools, honestly, I think it’s too much. I don’t think that function would really be helpful. It might be more of a distraction, or it might not even get used.

Participant 12

### User Experience and Potential Barriers to Using Smart Glasses

#### Overall User Experience

Overall, participants praised the user-friendly design of our application, finding it intuitive and easy to navigate. They expressed confidence in using the application with minimal training, as 1 participant stated:

Overall, it was pretty intuitive for me to use. I could see myself using it as is.

Participant 24

In addition, there is a consensus among almost all the participants that the smart glass application has great potential to improve communication efficiency and care coordination between distributed emergency care teams, as illustrated in the following 2 quotes:

It might be helpful because [when I] turn on the camera, they [physicians] would be able to see what we’re seeing, instead of us just having to, you know, dictate that over radio. So I think that’s kind of a big feature that would be useful there. I think more information would be passed along, compared to just how we do it right now.

Participant 41

It helps being able to give a heads up to the trauma team, so they know what to expect. Or even something like, let’s say it was a stroke. And you’ve got the neuro team waiting on standby to see if it’s really a stroke or not. It just gives so much more [information] and a clearer picture because you can actually show it [the patient] to them. I think that’s really cool.

Participant 12

The SUS rating improved from a mean of 74.3 (SD 11.3) in the first phase of usability evaluation (classified as *good* usability [52]) to a mean of 80.3 (SD 13.1) in the second phase of usability evaluation (classified as *excellent* rating [52]). Moreover, users’ willingness to use the system in the future increased from a mean of 4.42 out of 5 (SD 0.65) in phase 1 to a mean of 4.63 out of 5 (SD 0.62) in phase 2. Other user ratings, such as the application’s fit with the EMS workflow and its ease of navigation, remained consistently high across both phases. This notable positive user feedback demonstrated the continuous enhancement of our system’s design and usability through 2 phases.

The statistical analysis showed a significant difference in SUS ratings between EMS providers operating in urban areas (median 85, IQR 76-94) and those in rural areas (median 72.5, IQR 66-83; Mann-Whitney *U*=43; *P*=.17). Moreover, we observed a significant difference in SUS ratings between paramedics (median 72.5, IQR 70-80) and EMTs (median 85, IQR 74-98; Mann-Whitney *U*=44.5; *P*=.13). However, no significant difference was found in the ratings of different system features among participants with varying characteristics. Altogether, these findings imply that providers in urban areas and those with comparatively less training in treating patients in critical conditions perceived the smart glass application as more useful and user-friendly.

#### Potential Barriers to Adopting Smart Glasses in EMS Work

##### Overview

Despite the promising benefits of smart glasses in enhancing prehospital communication (eg, reducing the risk of miscommunication), our participants raised several potential challenges and concerns about adopting this technology in their work practice. As general issues related to the use of smart glasses—such as comfort, device durability, and ergonomic concerns—have been extensively reported in our prior studies [19,53], this paper specifically focuses on issues related to using smart glasses for prehospital communication. To better organize these challenges, we have categorized them into 3 groups: technical factors, environmental factors, and workflow integration considerations.

##### Technical Factors

A common concern about smart glasses is their reliance on high-speed network connectivity to establish video calls with remote care teams. Participants emphasized the necessity of ensuring stable and high-bandwidth internet connections, especially in areas with limited network coverage such as rural areas or subways. When the internet is unavailable, using smart glasses becomes impractical, as 1 participant explained:

I feel like it may be an issue with a lot of places we go around, such as some buildings and rural areas. So, it [internet] can be spotty. That even seems to be an issue with the current phones. It [stable internet connection] is critical for using smart glasses in the field.

Participant 13

Furthermore, EMS providers may be dispatched for search and rescue tasks that can extend over long durations. In such scenarios, the device’s battery life becomes crucial to ensuring uninterrupted communication between EMS providers and remote experts. One participant voiced the following concern:

Another concern for me is battery life. Especially since I can see it being useful in long-term rescue scenarios that can take time.

Participant 6

##### Environmental Factors

The dynamic and noisy setting of EMS work poses challenges for effective communication. Our participants expressed concerns about their ability to hear remote physicians clearly through the speakers of the smart glasses. One participant explained as follows:

In the back of an ambulance, it can be very loud, with sirens, horns, driving through town, hitting bumps, and everything rattling back there. Additionally, the monitor is beeping at you. So, I just want to make sure I’d be able to hear it.

Participant 42

##### Workflow Integration Considerations

Participants also discussed the workflow-related considerations associated with implementing the system. To fully use smart glasses, a counterpart system needs to be deployed in both OLMC offices and hospitals’ ED departments, requiring significant integration efforts:

You guys would have to integrate [the application] into every single notification system. Because right now, the notification system is kind of just like a red phone that’s in the emergency room.

Participant 35

Moreover, implementing this new system in receiving care teams may impact their workflow, potentially necessitating the assignment of a dedicated person to answer video calls. One participant highlighted this barrier:

You need internal systems in the hospital to function properly. You need your hospital to know that they’re going to be receiving this [video call and have someone ready to handle that one as it happens.

Participant 19

Finally, the EMS system in the United States is complex, involving multiple stakeholders beyond EMS agencies and hospitals, such as state and city regulators, insurance companies, etc. This complexity may present considerable challenges to the widespread adoption of smart glass technology, as 1 participant noted:

It’s a huge task to get this integrated with the billing service, ambulance services, software vendors, and many other things. It would also be quite an effort for every one of your partner hospitals to have an integrated video platform for communication. I think it’s going to be interesting to see how that plays out.

Participant 36

## Discussion

### Principal Findings

To the best of our knowledge, this study is the first of its kind that uses a user-centered design approach to explore the appropriate design of smart glasses in facilitating communication and care coordination between prehospital and remote care providers. Our approach is deeply rooted in PD, which promises to create user-friendly clinical systems and thoroughly addresses user needs [22,29,30,38]. In addition, our iterative design process involved 43 distinct EMS providers with diverse characteristics (eg, fire-based vs hospital-based agencies and urban vs rural areas). This approach is beneficial, as it can significantly increase the generalizability of our findings, effectively identify and address various user needs, and ensure that providers with different roles and occupations (eg, paramedics vs EMTs) can equally voice their needs and concerns.

Our work revealed that the prehospital communication process encompasses 2 critical activities: EMS providers reaching out to OLMC physicians for guidance and medical approval (eg, approving medication administration and determining patient destination) and notifying the receiving hospital about patient arrival. A significant challenge in both activities arises from the limitations of current communication mechanisms (eg, radio or phone) in conveying contextual patient information, often resulting in lengthy verbal descriptions or even miscommunication. Moreover, the shortage of OLMC physicians frequently causes delays in connecting with EMS providers, adversely affecting the efficiency and promptness of patient care in the field. Finally, the communication between EMS and receiving ED teams involves multiple layers, leading to unnecessary complexity in the process. Establishing a direct EMS-ED communication link was recognized as an essential need, as it holds the potential to minimize the chances of miscommunication during critical situations.

These findings, as well as specific user requirements elicited through design workshops and usability testing, informed our system design. The final version of our application incorporates several key features: (1) a video call functionality to contact OLMC physicians, allowing EMS providers to convey contextual patient information; (2) an option to indicate the call severity and priority to expedite the connection with OLMC physicians; (3) a direct video-based communication link with the ED team at the receiving hospital, supplemented with essential information such as ETA and the hospital’s special care capabilities, facilitating effective communication and aiding in patient destination decision-making; (4) an option to share critical, multimedia patient information (eg, photos, demographics, treatments, and vital signs) with remote care teams, augmenting the video call and providing comprehensive details for more informed medical decisions; (5) integration of touchless interaction methods, enabling hands-free operation and reducing the likelihood of cross contamination, a crucial aspect given the nature of EMS work.

The ratings given by the participants for our application showed a notable increase from phase 1 to phase 2, indicating continuous improvement in our system design and increased user acceptance of our application. Throughout the study, the study participants consistently highlighted the great potential and benefits of using smart glasses in prehospital communication, such as enhancing communication efficiency and care coordination between distributed emergency care teams. Despite this, the participants expressed a few concerns related to technical factors, environmental challenges, and workflow integration issues. These valuable insights provide important considerations for the further refinement and implementation of smart glasses in real-world settings.

### Study Implications and Future Directions

The effective use of smart glasses by EMS providers hinges on their access to a high-bandwidth cellular network to establish quality video and audio calls with remote care teams. However, EMS providers often find themselves in areas with limited signal coverage, presenting challenges for the effective use of smart glasses. Not surprisingly, the issue of reliable internet access for the field deployment of smart glasses was raised as a major concern by many participants and has been reported in previous studies [19,53]. A potential solution lies in harnessing the capabilities of 5G technology to enhance the network connectivity of smart glasses. In addition, ongoing initiatives to establish a dedicated broadband network for first responders [54] promise to address the connectivity issues encountered by EMS providers.

In addition, EMS providers mentioned several other potential barriers (eg, battery life and environmental constraints) to adopting smart glass technology in real practice. Compared to the first generation of smart glasses released almost a decade ago, recent advancements in smart glass hardware have the potential to address some of the users’ concerns. For instance, the device we used—the Vuzix M400—is waterproof, has up to 12 hours of battery life, and can operate in a wide range of temperatures (from −20 °C to 45 °C). These hardware advancements can make the smart glass device adaptable to the EMS setting.

The diverse characteristics of our EMS participants allowed us to explore the disparities in their perceptions of smart glasses. This study revealed that providers in urban areas, especially those with less experience in treating patients in critical conditions, perceived the smart glass application as more beneficial and user-friendly. These insights underscore the fact that user perspectives and preferences concerning smart glasses can differ based on location and occupation. Essentially, the notion of a universal solution (eg, “one size fits all”) may not be applicable when designing smart glass applications for EMS providers. Thus, a smart glass–based telemedicine application must be adaptable to meet the distinct needs of EMS agencies in various locations.

An integral aspect of our smart glass application is its capability to share patient information with remote care teams. To realize this feature, the smart glass application needs to be integrated with the existing systems used by EMS providers, such as vital signs monitors and electronic health record systems. However, implementing these integrations could face real-world challenges, primarily due to persistent issues in health system interoperability [55]. Future work could explore viable technical and organizational strategies to effectively integrate smart glasses with medical devices and systems (eg, collaborating with electronic health record vendors and adopting the Fast Healthcare Interoperability Resources standard [56] to enhance their interoperability). In addition, it is critical to ensure that data transmission between smart glasses and medical systems complies with the rules and regulations of the Health Insurance Portability and Accountability Act [57].

This study primarily focused on the design of a smart glass application tailored for EMS providers; however, there are other stakeholders that are critical for the successful implementation of this technology. For example, fully realizing the potential of smart glasses also requires developing and deploying corresponding telemedicine applications for remote care teams, such as OLMC physicians and hospital ED teams. Our forthcoming work involves engaging with these key stakeholders to determine the optimal design and implementation strategy for their respective telemedicine systems. In addition, as emphasized by our participants, other stakeholders beyond care providers, such as EMS regulators and insurance companies, play a critical role in regulating EMS work and affecting technology adoption. Therefore, future research should explore the viewpoints of these additional stakeholders. Attaining “buy-in” from the leadership of these key stakeholders is a critical determining factor in adopting smart glasses [53].

Finally, it is worth noting that technology solutions such as smart glasses or other telemedicine systems can only partially address the longstanding challenges in the prehospital communication process. Some challenges are systemic and must be tackled at the organizational and policy levels. For example, the shortage of OLMC physicians is a major reason for delayed responses to EMS calls and requests. Although our smart glass design includes features to ensure that critical calls are answered promptly by OLMC physicians, it cannot completely resolve issues stemming from the lack of OLMC physicians.

### Limitations

This study has certain limitations that should be acknowledged. First, we did not evaluate the application in a natural working environment, meaning we have not fully assessed how smart glasses are used in real practice. To address this, we plan to conduct system testing in near–real-life simulations in the future to investigate the effectiveness of smart glasses in enhancing patient care in the field. Second, although we identified and designed a feature for expedited connection with OLMC physicians, we did not elicit the perspectives of OLMC physicians on its usefulness. To address this gap, we plan to seek feedback and insights from OLMC physicians in future studies, with a specific focus on the call priority and video call features. Third, our study was conducted in the United States, and the attributes and dynamics of prehospital communication here may differ from those in other countries. It is likely that the smart glass application we developed is not applicable to the EMS contexts in different nations. Nonetheless, the design insights and user perspectives on smart glasses could provide valuable guidance for researchers in other regions developing smart glass solutions for emergency care teams. Finally, the evaluation of the smart glass application focused primarily on usability and user perceptions, without assessing the impact of our application on clinical outcomes. In our future work, we will conduct simulations to test the effectiveness of smart glasses in improving the prehospital communication process and whether using smart glasses could help make better decisions and reduce medical errors.

### Conclusions

This paper presents a 2-year long, user-centered design research project on a smart glass application aimed at enhancing prehospital communication. Our work illustrated critical features deemed useful by EMS providers, such as video call functionality for expedited OLMC contact, direct video-based communication with ED teams, multimedia patient information sharing, and touchless interaction mechanisms. Rooted in PD, the study has yielded invaluable insights into leveraging smart glasses for enhancing communication and care coordination in the dynamic prehospital environment. Our work reaffirms the critical value of user-centered design in health care technology innovation. The findings and lessons learned from our study are poised to guide future work on the implementation and integration of smart glasses into prehospital care.

## Appendix

Multimedia Appendix 1 Participant characteristics.

Multimedia Appendix 2 Participatory design workshop protocol.

Multimedia Appendix 3 Usability testing protocol.

## Abbreviations

ED: emergency department
EMS: emergency medical service
EMT: emergency medical technician
ETA: estimated time of arrival
HIT: health information technology
NDK: Native Developer Kit
OLMC: online medical control
PD: participatory design
STEMI: ST elevation myocardial infarction
SUS: System Usability Scale

## Data Availability

The datasets generated during and analyzed during this study are not publicly available due to institutional review board restrictions and the need to protect human participants’ confidentiality, but are available from the corresponding author on reasonable request.
