# Supplementary material for: A Smart Glass Telemedicine Application for Prehospital Communication: User-Centered Design Study
Source: J Med Internet Res. 2024 Nov 29;26:e53157. doi: 10.2196/53157 (PMC11645503; doi:10.2196/53157)
Supplement: Multimedia Appendix 1 [file jmir_v26i1e53157_app1.docx]

Table 1. Participant characteristics

| ID | Characteristics of Affiliated EMS Agency | Occupation | Years of Experience | Participated Sessions |
| --- | --- | --- | --- | --- |
| 1 | Urban area, hospital-based | Paramedic & EMS Director | 44 years | All studies |
| 2 | Urban area, hospital-based | EMT | 3 years | Phase 1 PD workshop |
| 3 | Urban area, hospital-based | EMT | 10 years | Phase 1 PD workshop |
| 4 | Urban area, hospital-based | EMT | 5 years | Phase 1 PD workshop |
| 5 | Urban area, hospital-based | Paramedic | 10 years | Phase 1 and Phase 2 PD workshops |
| 6 | Urban area, hospital-based | Paramedic | 20 years | Phase 1 and Phase 2 PD workshops |
| 7 | Urban area, hospital-based | Paramedic | 27 years | Phase 1 PD workshop |
| 8 | Urban area, hospital-based | Paramedic | 3 years | Phase 1 PD workshop |
| 9 | Urban area, hospital-based | EMT | 6 years | Phase 1 PD workshop |
| 10 | Urban area, hospital-based | EMT | 2 years | Phase 1 PD workshop |
| 11 | Urban area, hospital-based | EMT | 5 years | Phase 1 PD workshop |
| 12 | Rural area, fire-based | Paramedic | 19 years | Phase 1 PD workshop and usability testing, Phase 2 usability testing |
| 13 | Rural area, fire-based | Paramedic | 10 years | All studies |
| 14 | Rural area, fire-based | Paramedic | 9 years | Phase 1 and Phase 2 PD workshops |
| 15 | Rural area, fire-based | Paramedic | 16 years | Phase 1 PD workshop |
| 16 | Urban area, hospital-based | EMT |  | Phase 1 usability testing |
| 17 | Urban area, hospital-based | EMT |  | Phase 1 usability testing |
| 18 | Urban area, hospital-based |  |  | Phase 1 usability Testing |
| 19 | Urban area, hospital-based | EMT | Less than 1 year | Phase 1 usability testing, Phase 2 PD workshop |
| 20 | Urban area, hospital-based |  | Less than 1 year | Phase 1 and Phase 2 usability testing |
| 21 | Urban area, hospital-based |  | Less than 1 year | Phase 1 usability testing |
| 22 | Urban area, hospital-based |  |  | Phase 1 usability testing |
| 23 | Rural area, fire-based |  | 5 years | Phase 1 usability testing |
| 24 | Rural area, fire-based |  | 2 years | Phase 1 usability testing |
| 25 | Rural area, fire-based |  | 1 year | Phase 1 usability testing |
| 26 | Urban area, hospital-based | EMT | 10 years | Phase 2 PD workshop |
| 27 | Urban area, hospital-based | Paramedic | 29 years | Phase 2 PD workshop |
| 28 | Urban area, hospital-based | EMT | 25 years | Phase 2 PD workshop |
| 29 | Urban area, hospital-based | Paramedic | 19 years | Phase 2 PD workshop |
| 30 | Rural area, fire-based | Paramedic | 5 years | Phase 2 PD workshop |
| 31 | Rural area, fire-based | EMT | 14 years | Phase 2 PD workshop |
| 32 | Urban area, hospital-based | EMT | 5 years | Phase 2 usability testing |
| 33 | Urban area, hospital-based | EMT | 3 years | Phase 2 usability testing |
| 34 | Urban area, hospital-based | Paramedic | 13 years | Phase 2 usability testing |
| 35 | Urban area, hospital-based | Paramedic | 4 years | Phase 2 usability testing |
| 36 | Urban area, hospital-based | Paramedic | 4 years | Phase 2 usability testing |
| 37 | Urban area, hospital-based | EMT | 3 years | Phase 2 usability testing |
| 38 | Urban area, hospital-based | EMT | 6 years | Phase 2 usability testing |
| 39 | Urban area, hospital-based |  | 5 years | Phase 2 usability testing |
| 40 | Rural area, fire-based | Paramedic | 25 years | Phase 2 usability testing |
| 41 | Rural area, fire-based | EMT | 6 years | Phase 2 usability testing |
| 42 | Rural area, fire-based | EMT | 8 years | Phase 2 usability testing |
| 43 | Rural area, fire-based | Paramedic | 25 years | Phase 2 usability testing |
