# Supplementary material for: A Smart Glass Telemedicine Application for Prehospital Communication: User-Centered Design Study
Source: J Med Internet Res. 2024 Nov 29;26:e53157. doi: 10.2196/53157 (PMC11645503; doi:10.2196/53157)
Supplement: Multimedia Appendix 2 [file jmir_v26i1e53157_app2.pdf]

TODAY'S WORKSHOP

4m

- About our research team & project
- Our goals for today
  - Design a hands-free smart glass application to support pre-hospital communication
  - We need your input!

TODAY'S SCHEDULE

1m

Time	Activity
	Introductions (15min)
	Storytelling (15min)
	Individual Designs & Sharing (30min)
	Group Designs (25min)
	Discussion (30min)
	Wrap-up (5min)

INTRODUCTIONS

5m

- Participant introductions:
  - Your name
  - Experience
  - Something about yourself

YOUR PARTICIPATION

5m

- Your participation in this workshop is voluntary and you may choose to leave at any time.
- We will be recording today's session for data analysis purposes only.
- Your identity will not be associated with any data or used in any subsequent presentations or publications.

Do we have your permission to start recording?

STORYTELLING

10m

Please tell us about your experience with pre-hospital communication, specifically in sharing information with the receiving care team.

DESIGN PROBLEM: Inefficient Pre-Hospital Communication 3m

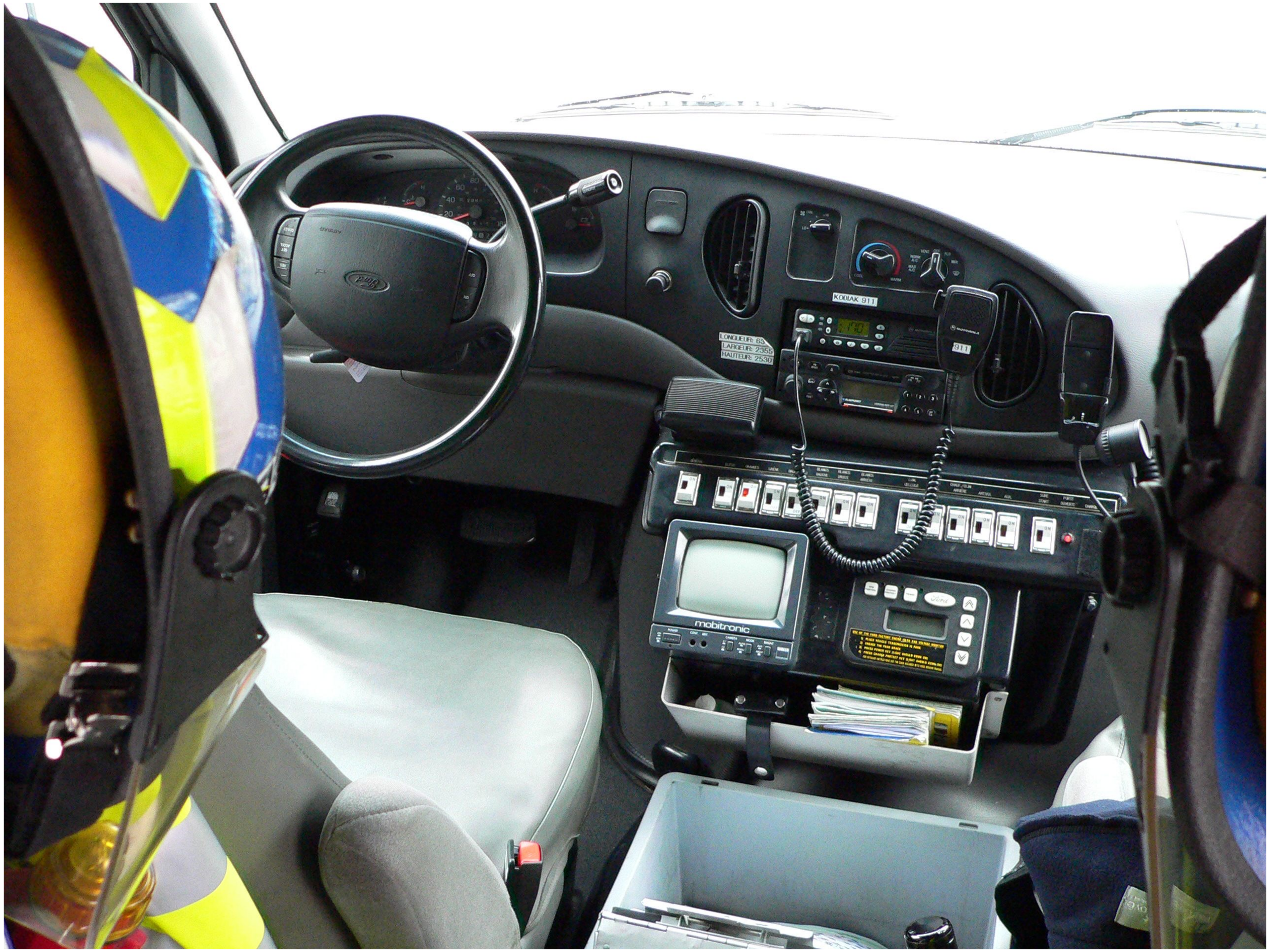

DESIGN PROBLEM (Cont'd):
Initial ideas… 2m

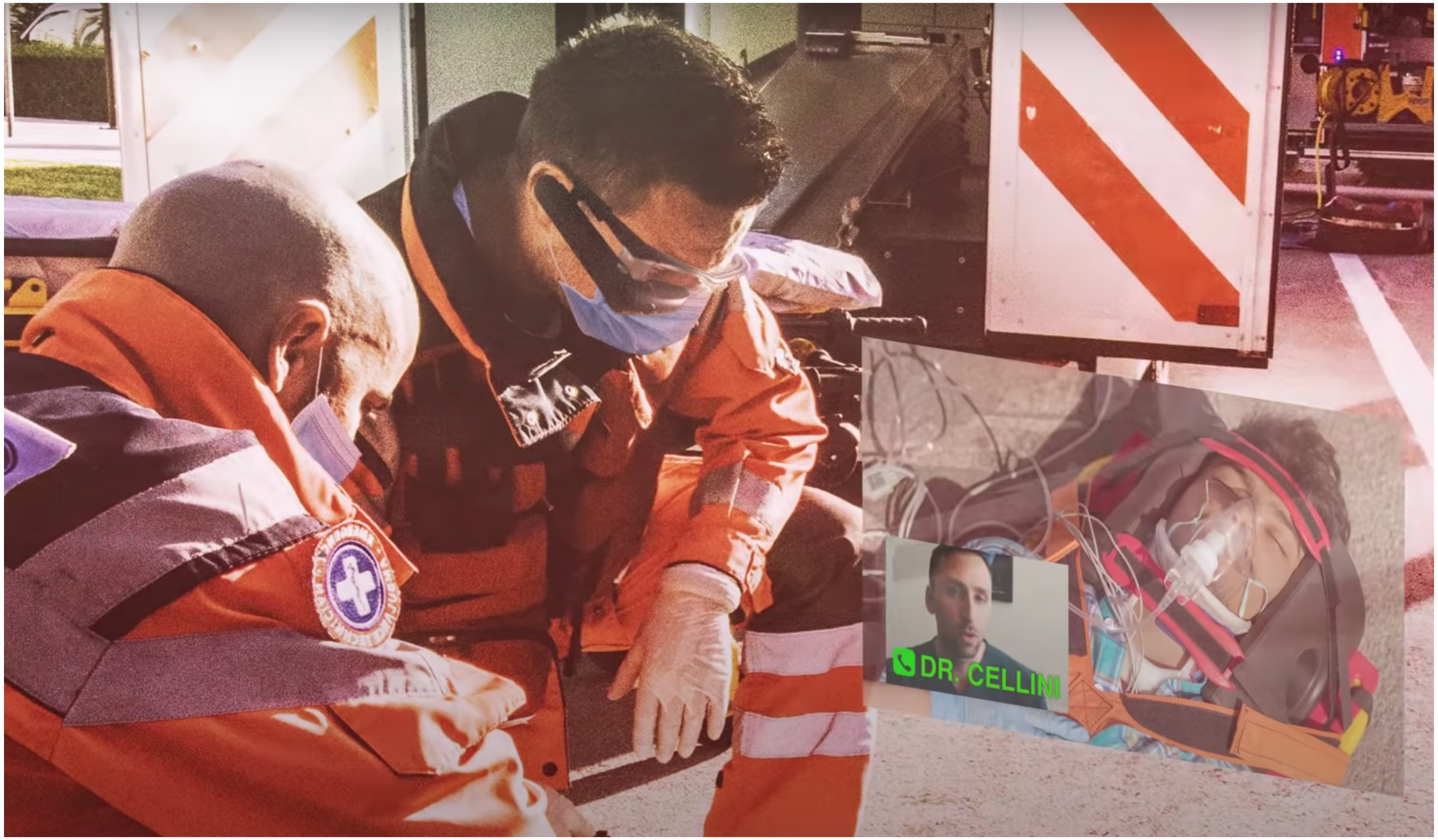

INDIVIDUAL DESIGN: FEATURES & INTERFACES

20m

- What **features** do you want to see?
- What types of **clinical content** do you want to show via smart glasses?
- How do you want the smart glass **interface** to look?

- Use the **printed glass screens** to create the interfaces of the smart glass

INDIVIDUAL DESIGN: SHARING

10m

Each participant shares their design.

Talk a little bit about the features you included and why.

5 MINUTE BREAK

GROUP DESIGN

25m

- Now thinking back on your individual designs, discuss some of the features and functionalities you think should be included in the smart glass.

- Please design an application that everyone agrees upon and then demonstrate your design as a group.

GROUP DISCUSSION 10m

Interactions
Which interaction method do you like best? & Why? (Demonstrate & Rank a set of hands-free interaction methods)

Human Computer Interaction
Do you think wearing the smart glass will impede your work in anyway? Do you think you'll feel comfortable wearing your smart glass?

People
What other stakeholders can we talk to to get more ideas for developing and deploying this system?

GROUP DISCUSSION (Cont'd) 10m

Workflow
To what extent do you think this can affect your current work practice? Would EMTs & paramedics need to modify current workflow to use the device?

Internal Policies and Procedures
Are there any current policies or procedures of your organization that can affect the adoption of the technology?

External Rules, Regulations, and Pressures
Do you think external rules like HIPAA can affect the adoption? Based on your experience, can you tell us how we can design this technology to comply to existing rules and regulations?

GROUP DISCUSSION (Cont'd) **10m**

System Measurement and Monitoring
Can you share ways we can measure the effectiveness and usefulness of this system?

Concerns
Do you have any concerns about using the smart glass technology (e.g., hardware, software, privacy, data security, ergonomics)?

What kinds of potential barriers may affect its adoption (e.g., integration with workflow, etc.)?

WRAP-UP: FEEDBACK

5m

- How was your experience today?
- What can we improve?

THANK YOU!

If you have any other questions or comments, please feel free to contact us:

Dr. Zhan Zhang
zzhang@pace.edu
