# Supplementary material for: A Smart Glass Telemedicine Application for Prehospital Communication: User-Centered Design Study
Source: J Med Internet Res. 2024 Nov 29;26:e53157. doi: 10.2196/53157 (PMC11645503; doi:10.2196/53157)
Supplement: Multimedia Appendix 3 [file jmir_v26i1e53157_app3.pdf]

Usability Testing with EMS Providers

Protocol

1. Evaluators introduce themselves and the goal of the study, review participant's rights, and administer the informed consent form (10 min).
2. Evaluators introduce the smart glass device and train/show the participant how to use it (20 min).
3. Participant is asked to complete the tasks using the think aloud protocol (20 min).
4. Participant completes a survey and evaluator prompts participant with discussion questions; participant can annotate a paper version of the system as necessary (10 min).

Tools & Materials

Hardware

- Smart Glasses (Vuzix M400)
- Tablet
- Laptop
- Video/audio Recorders
- Power Bank

Software

- Screen Recorder
- Zoom for recording participant's actions and reactions

Other

- Printouts (consent form, protocol, questionnaire)
- Notebook and pen for notes
- Paper version of the application
- Index cards for voice commands

Introduction

- Thank you for coming today and taking time out of your schedule to talk with us.
- We're a team of researchers from Pace University.
- The overall goal of our project is to facilitate apre-hospital communication in the field via the hands-free smart glass technology.
- We have been working with both paramedics and EMTs designing the features of this system.
- We have created a partially functional system with several features implemented, i.e., offering visual-based communication between EMS providers and remote physicians.
- Today we'd like to ask you to test out some major features of our system, then complete a survey, and wrap-up with some discussions.

  Some important things to remember throughout the study:
- If you are having problems completing some of the tasks, please bear in mind that the purpose of the test is to uncover these issues which exist with the interface so we can make changes to improve the experience. Also, today we will be using a partially implemented system so some functionalities might not work as expected.
- This is a test of the user interface and major features of this system, and not a test of you. You are the domain expert, and we want to hear your ideas about how we can improve the design of our systems.
- During the test, we would like to ask you to 'think aloud', meaning please tell us your exact thoughts of each step when you try to achieve something, and how you expect to achieve this with the options available to you.
- Try to think about completing the tasks as you would if you were doing the task in real practice.

Participant Rights

- [Administer the informed consent form]
- [While the participant is reviewing the consent form, go through the following bullet points]
- Just to cover your rights as a participant for your protection.
- Your participation in this session is voluntary and you may choose to leave at any time.
- We will be audio and video recording today's session for data analysis purposes only. We may also take photographs to document the evaluation process.
- Your identity will not be associated with any data or used in any subsequent presentations or publications.
- Do you have any questions?
- [Ask them to sign the consent form]

Training

- Evaluator first goes over the **main features** (Figma) [10 min].
- Then show how to interact with the device using **hand gestures, voice commands, and buttons**. After showing one method, ask the participant to try out that method using the dummy app [participants can take up to 10 min].
- Once the participant feels they are ready to go, then move on to the next phase.

Testing Session

[For each part, randomize the order of different interaction modalities: hand gesture, voice command, buttons]
[Complete all tasks with one modality then repeat the tasks with another modality]

Task 1: You want to call the centralized telemetry to discuss a pediatric traumatic brain injury patient. Once you are connected, turn on the camera to show the patient.

Task 2: The EMS team is ready to transport the patient to the first hospital displayed in the list. You want to 1) call the receiving hospital to notify them and 2) to share the collected patient information with them.

[Administer survey for this application]

EMS Survey - Teleconsultation

**Please indicate your regular role:**<br>☐ Paramedic ☐ EMT ☐ Other _________
**Indicate which EMS agency you are part of:** _________
**Number of years' experience in this role:** _________

Questionnaire

Please think aloud and explain your reasoning behind the rating.
Annotate the paper version, if necessary, to demonstrate what you mean.

Features and Design

1. The feature to video call with OLMC is useful.

1	2	3	4	5
Strongly Disagree		Neutral		Strongly Agree

2. The feature to video call with hospital is useful.

1	2	3	4	5
Strongly Disagree		Neutral		Strongly Agree

3. The feature to share info with hospital while calling is useful.

1	2	3	4	5
Strongly Disagree		Neutral		Strongly Agree

4. The feature to indicate patient category/symptoms before calling OLMC is necessary.

1	2	3	4	5
Strongly Disagree		Neutral		Strongly Agree

5.	I think it is easy to use hand gestures to control the device.				
	1<br>Strongly Disagree	2	3<br>Neutral	4	5<br>Strongly Agree
6.	I think it is easy to use voice commands to control the device.				
	1<br>Strongly Disagree	2	3<br>Neutral	4	5<br>Strongly Agree
7.	I would rather use touchpad than voice commands and hand gestures.				
	1<br>Strongly Disagree	2	3<br>Neutral	4	5<br>Strongly Agree
8.	The layout of the buttons on the screen is intuitive and easy to navigate.				
	1<br>Strongly Disagree	2	3<br>Neutral	4	5<br>Strongly Agree
9.	The glass screen does not block my vision.				
	1<br>Strongly Disagree	2	3<br>Neutral	4	5<br>Strongly Agree
10.	It is easy to recover from errors.				
	1<br>Strongly Disagree	2	3<br>Neutral	4	5<br>Strongly Agree
11.	The system fits into my workflow.				
	1<br>Strongly Disagree	2	3<br>Neutral	4	5<br>Strongly Agree

12. I am willing to use the system to communicate with telemetry or hospital in the future.

1	2	3	4	5
Strongly Disagree		Neutral		Strongly Agree

System Usability Scale

1. I think that I would like to use this system frequently.

1	2	3	4	5
Strongly Disagree		Neutral		Strongly Agree

2. I found the system unnecessarily complex.

1	2	3	4	5
Strongly Disagree		Neutral		Strongly Agree

3. I thought the system was easy to use.

1	2	3	4	5
Strongly Disagree		Neutral		Strongly Agree

4. I think that I would need the support of a technical person to be able to use this system.

1	2	3	4	5
Strongly Disagree		Neutral		Strongly Agree

5. I found the various functions in this system were well integrated.

1	2	3	4	5
Strongly Disagree		Neutral		Strongly Agree

6. I thought there was too much inconsistency in this system.				
1<br>Strongly Disagree	2	3<br>Neutral	4	5<br>Strongly Agree
7. I would imagine that most people would learn to use the system very quickly.				
1<br>Strongly Disagree	2	3<br>Neutral	4	5<br>Strongly Agree
8. I found the system very awkward to use.				
1<br>Strongly Disagree	2	3<br>Neutral	4	5<br>Strongly Agree
9. I felt very confident using the system.				
1<br>Strongly Disagree	2	3<br>Neutral	4	5<br>Strongly Agree
10. I needed to learn a lot of things before I could get going with this system.				
1<br>Strongly Disagree	2	3<br>Neutral	4	5<br>Strongly Agree

- End of Survey -

Post-Testing Interview Questions

Open-Ended Questions
1. Please describe your overall impression of this part of smart glass application.
2. What did you like and dislike the most when using this part of smart glass application?
3. How do you feel about the layouts in this part of smart glass design? Is there any icon that you think should be larger or smaller, or in other places?
4. What changes or new features do you hope to see in this part of smart glass application?
5. Any other concern regarding using this smart glass application in practice?
6. When using the application, how do you rank the three interaction methods from easiest to use to hardest to use? And why?
7. Any other comments, thoughts, or ideas that you would like to share?

Note: This study protocol is shared as part of our publication: Zhan Zhang, Enze Bai, Yincao Xu, Aram Stepanian, Mustafa Ozkaynak, Jared Kutzin, Kathleen Adelgais. User-Centered Design of a Smart Glass Telemedicine Application for Prehospital Communication. Journal of Medical Internet Research.
